# Lesbian, Gay, & Bisexual Health Inequities and Financial Burden: Health & Retirement Study Insights

**DOI:** 10.1093/geroni/igaf122.3618

**Published:** 2025-12-31

**Authors:** Jennifer May, Hanna Smyles, Swann Adams, Matthew Lohman

**Affiliations:** University of South Carolina, Columbia, South Carolina, United States; University of South Carolina- College of Nursing, Columbia, South Carolina, United States; University of South Carolina, Columbia, South Carolina, United States; University of South Carolina, Columbia, South Carolina, United States

## Abstract

Older lesbian, gay, and bisexual (LGB) adults often face distinct health and social challenges compared to their heterosexual counterparts. This study leverages the nationally representative Health and Retirement Study (HRS) to examine health inequities and financial burden among LGB older adults compared to their non-LGB counterparts in the United States. Using the harmonized RAND dataset from waves 2016, 2018, and 2020, we compared self-identified LGB and non-LGB adults aged 51 and older, including respondents under 50 living in age-eligible households. Weighted descriptive statistics were calculated for prescription drug use, self-rated health, chronic conditions, and hospital nights, with differences by LGB status assessed using chi-square tests. Across all waves, prescription drug use, chronic conditions, and hospital nights did not significantly differ by LGB status. However, consistent and statistically significant differences in self-rated health were observed. LGB adults were more likely to report fair or poor health than non-LGB peers (e.g., in 2020, fair; 27.9% LGB vs 20.2% non-LGB; poor: 6.0% LGB vs 4.6% non-LGB; p < 0.0001), with fewer LGB respondents reporting very good or excellent health. These gaps in subjective health status persisted over time despite similar patterns of healthcare utilization and chronic disease burden. Findings highlight the need for clinicians and policymakers to be aware of and address the distinct challenges of older sexual minorities through targeted health promotion, culturally competent care, and inclusive policy initiatives.

